# Peripheral Hemolysis in Relation to Iron Rim Presence and Brain Volume in Multiple Sclerosis

**DOI:** 10.3389/fneur.2022.928582

**Published:** 2022-06-29

**Authors:** Nik Krajnc, Gabriel Bsteh, Gregor Kasprian, Tobias Zrzavy, Barbara Kornek, Thomas Berger, Fritz Leutmezer, Paulus Rommer, Hans Lassmann, Simon Hametner, Assunta Dal-Bianco

**Affiliations:** ^1^Medical University of Vienna, Department of Neurology, Vienna, Austria; ^2^Faculty of Medicine, University of Ljubljana, Ljubljana, Slovenia; ^3^Medical University of Vienna, Biomedical Imaging and Image-Guided Therapy, Vienna, Austria; ^4^Center for Brain Research, Medical University of Vienna, Vienna, Austria; ^5^Division of Neuropathology and Neurochemistry, Department of Neurology, Medical University of Vienna, Vienna, Austria

**Keywords:** iron rim, hemolysis, multiple sclerosis, disease progression, brain volume

## Abstract

**Background:**

Iron rim lesions (IRLs) represent chronic lesion activity and are associated with a more severe disease course in multiple sclerosis (MS). How the iron rims around the lesions arise in patients with MS (pwMS), and whether peripheral hemolysis may be a source of iron in rim associated macrophages, is unclear.

**Objective:**

To determine a potential correlation between peripheral hemolysis parameters and IRL presence in pwMS.

**Methods:**

This retrospective study included pwMS, who underwent a 3T brain MRI between 2015 and 2020 and had a blood sample drawn at ± 2 weeks. Patients with vertigo served as a control group.

**Results:**

We analyzed 75 pwMS (mean age 37.0 years [SD 9.0], 53.3% female) and 43 controls (mean age 38.3 years [SD 9.8], 51.2% female). Median number of IRLs was 1 (IQR 4), 28 (37.3%) pwMS had no IRLs. IRL patients showed significantly higher Expanded Disability Status Scale (EDSS) compared to non-IRL patients (median EDSS 2.3 [IQR 2.9] vs. 1.3 [IQR 2.9], *p* = 0.017). Number of IRLs correlated significantly with disease duration (*r*_s_ = 0.239, *p* = 0.039), EDSS (*r*_s_ = 0.387, *p* < 0.001) and Multiple Sclerosis Severity Scale (MSSS) (*r*_s_ = 0.289, *p* = 0.014). There was no significant difference in hemolysis parameters between non-IRL, IRL patients (regardless of gender and/or disease type) and controls, nor between hemolysis parameters and the number of IRLs. Total brain volume was associated with fibrinogen (*β*= −0.34, 95% CI −1.32 to −0.145, *p* = 0.016), and absolute cortical and total gray matter volumes were associated with hemoglobin (*β* = 0.34, 95% CI 3.39–24.68, *p* = 0.011; *β* = 0.33, 95% CI 3.29–28.95, *p* = 0.015; respectively).

**Conclusion:**

Our data do not suggest an association between hemolysis parameters and IRL presence despite a significant association between these parameters and markers for neurodegeneration.

## Introduction

Multiple sclerosis (MS) is a chronic inflammatory disease of the central nervous system (CNS), which leads to focal demyelination in the gray and white matter (WM) (1, 2). In the early relapsing stage of the disease, acute inflammation and blood-brain barrier (BBB) disruption is reflected by gadolinium (Gd) enhancing lesions in MRI. Later, chronic active inflammation behind the BBB can be detected by MRI *via* susceptibility-weighted imaging (SWI), R2^*^ or QSM as a rim of iron-laden microglia and macrophages around the FLAIR-hyperintense lesion (3–6). These so-called iron rim lesions (IRLs) or paramagnetic rim lesions are a subset of chronic active lesions (3, 6–9), and occur in ~60% of people with MS (pwMS) irrespective of MS course peaking in the late relapsing-remitting MS and early secondary progressive MS stage (10). Approximately 30% of all WM lesions and about 40% of chronic active MS lesions have an iron rim (11). Their presence has been associated with a more severe disease course (12, 13). They display more pronounced black holes compared to non-IRLs, reflecting severe axonal loss and the absence of remyelination (4, 5), and they are associated with elevated serum neurofilament levels (sNfL) (14, 15) and brain atrophy rates (13, 14, 16). Recent work has shown that IRLs expand over time (3, 4, 17), while the iron rims themselves gradually diminish over an extended time period of about 7 years (17). Altogether, IRLs are now considered a potential biomarker for progression and chronic MS course with high neurodestructive potential (5, 18).

Generally, iron is known to accumulate in the human brain with age (19). On the one hand, it is important for normal cellular functions, biochemical reactions such as DNA, RNA, and proteins synthesis, and is involved in myelin synthesis (20, 21). On the other hand, iron is also cytotoxic due to free oxygen or nitrogen radical formation and is known to amplify demyelination, neurodegeneration, and oxidative damage in MS (8). Most cerebral iron is found in the substantia nigra and basal ganglia (22–24). An intact BBB protects the brain from fluctuations in systemic iron levels so that impaired iron homeostasis in the periphery has only minor effects on brain iron metabolism (25). Thus, concentrations of iron and iron-modulating proteins in the serum and cerebrospinal fluid (CSF) differ substantially (26). Since iron metabolism in terms of uptake, transport, and storage is not fully clarified (27), a better understanding of dysregulatory pathways of iron homeostasis will help elucidate the causes of iron accumulation and iron-mediated tissue damage in the brain. Recent advances in the field of iron-dependent lipid peroxidation leading to a cell death called “ferroptosis” have already provided valuable insights that are particularly relevant to the lipid-rich environment of the CNS (28).

Nevertheless, to date, no significant factor influencing the development of IRLs in the CNS has been identified, and it is still unclear why some pwMS have iron rims and others have not. We raise the question, whether peripheral hemolysis in pwMS under conditions of a chronically impaired BBB with potentially higher iron influx into the lesion may favor the formation of iron rims, which in turn are formed by phagocytes which protect brain tissue from free pro-oxidant Fe^2+^.

Iron is present in serum as both heme and non-heme iron. The former is used for metabolic processes, is highly concentrated in erythrocytes, and is liberated upon hemolysis. Liberated hemoglobin and its iron are bound by the serum protein haptoglobin to avoid its deleterious pro-oxidative effects (29). Main sources for iron-accumulation in the CNS are erythrocytes leaking through the BBB and their decay within the brain and spinal cord or the destruction of iron containing oligodendrocytes and myelin (4, 30). Already, early studies describe a macrocytosis and higher osmotic fragility of erythrocytes in pwMS than in healthy controls, and particularly more pronounced in patients with a relapse (31, 32). Also, a recent experimental study on iron overload found an increased fragility and macrocytosis of erythrocytes, low-grade hemolysis and a significant liberation of hemoglobin from erythrocytes (33). In addition, extracellular methemoglobin (metHb) was observed to cause oxidative damage to myelin components in the CNS after extravasation of blood from plaque veins into plaque tissue (34). This could eventually mean that iron release from myelin together with blood-derived iron amplifies neurodegeneration. Lewin et al. reported that elevated serum free hemoglobin (Hb) correlated with brain atrophy rate in people with secondary progressive MS (35). This has been attributed to chronic, low-grade intravascular hemolysis, which in turn is thought to lead to oxidative damage of oligodendrocytes by serum Hb after its passage through the impaired BBB. Nevertheless, intravascular hemolysis was not reflected by a lower total blood Hb level (35). Altogether, these data indicate that hemolysis may play a role in MS as a cofactor enhancing neurodegeneration. It is already known that elevated iron levels in postmortem brain SWI-MR images and iron deposition seen in histopathology correlate positively in pwMS (30, 36), but it is unknown whether peripheral serum parameters of hemolysis are associated with SWI-detected IRLs.

## Methods

### Patients

In this cross-sectional, retrospective study, 75 patients from the Vienna MS database (VMSD) (37) and 43 sex- and age-matched patients with peripheral vertigo as control group were included. All included control patients with peripheral vertigo were clearly clarified as not being centrally affected by our experts of our special outpatient clinic for vertigo based on clinical history, neurological status and imaging. Clinically definite MS was determined according to the established diagnostic criteria (38, 39). All pwMS met the following inclusion criteria: ≥18 years, availability of T1, FLAIR and SWI-based MRI scan at 3T, and a blood sample drawn at ±2 weeks from MRI. None of the patients had a diagnosed hemolytic disease. Data on expanded Disability Status Scale (EDSS) and Multiple Sclerosis Severity Scale (MSSS) according to Roxburgh et al. were obtained at the time of MRI (40), and clinical activity (relapses) was analyzed in a time period ±6 months from MRI. A severe relapse was defined as a relapse that required either treatment with steroids or hospitalization. Disease-modifying treatment (DMT) status was classified as following: (1) “no DMT” defined as patients receiving no DMT; (2) “moderately effective DMT” (M-DMT) defined as patients receiving either interferon-beta, glatiramer acetate, dimethyl fumarate, or teriflunomide; or (3) “highly effective DMT” (H-DMT) defined as patients receiving either natalizumab, fingolimod, alemtuzumab, cladribine, ocrelizumab or rituximab. MRI acquisition, including sequences such as FLAIR, T1 and SWI-based MRI for assessing lesions and brain volume (see below) and their analysis were performed at the Medical University of Vienna.

### Imaging Acquisition

All 3T MRI brain scans were performed on a Siemens Magnetom 3T MRI system, using a 64-channel radio frequency (RF) coil between January 1, 2015, and December 31, 2020. Isovoxel (1 mm3) 3DFLAIR (TR = 6,000 ms, TE = 288 ms, TI = 2,100 ms), T1 weighted images (TE = 2,16 ms, TR = 1,670 ms and flip angle = 15) with a gadolinium-based contrast administration and SWI sequences (TE = 40 ms, TR = 49,ms, image matrix = 224 × 256, slices = 80, slice thickness = 2 mm) were acquired consecutively.

### Evaluation of Lesions and Brain Volume

All supratentorial lesions of the periventricular, juxtacortical and deep white matter in the frontal, parietal and occipital lobes (41), and in the upper parts of the temporal lobes as well as infratentorial lesions of the cerebellum were analyzed in consensus by two independent raters (ADB, NK) highly experienced in MS imaging. IRLs were defined as FLAIR-hyperintense lesions that were partially or completely surrounded by a pronounced and distinct SWI-hypointense rim. The presence of the central plaque vein did not affect the iron rim evaluation. After both raters had made their decision, the unclear lesions were discussed together on the monitor and an agreement was reached. The inter-rater agreement before matching was 98.7%.

Volume of T1 lesions and total brain volume were automatically assessed using the MorphoBox prototype imaging software normalized for age from Siemens Healthineers (42). IRLs were considered valid if a hypointense SWI signal entirely or partially surrounded a hyperintense white matter lesion in FLAIR images. Patients were grouped for the presence of IRLs (no IRLs vs. ≥1 IRLs).

### Hemolysis Parameters

Hemolysis parameters included red blood cell (RBC) count, reticulocytes, Hb, hematocrit (Ht), potassium, iron, total bilirubin, free Hb, hemolysis index, lactate dehydrogenase, fibrinogen and aspartate transaminase levels. Blood samples were drawn and analyzed at the Department of Laboratory Medicine, Medical University of Vienna. The quality of blood samples was maintained with the usage of standardized protocols for their collection and storage.

### Ethics

The study was approved by the Ethics Committee of the Medical University of Vienna (EC 1599/2021).

### Statistics

Statistical analysis was performed using SPSS 26.0 (SPSS Inc, Chicago, IL, USA). Categorical variables were expressed in frequencies and percentages, continuous variables as mean and standard deviation (SD) or median and interquartile range (IQR) as appropriate. Continuous variables were tested for normal distribution by the Kolmogorov–Smirnov test. Univariate comparisons were done by chi-square test, independent *t*-test, Mann-Whitney *U*-test or Kruskal-Wallis test as appropriate.

Hemolysis parameters, clinical (EDSS, MSSS, disease course and duration) and paraclinical parameters [total brain volume, total gray matter (GM) and cortical volume, WM volume, total lesion volume], and the number of IRLs were first univariately analyzed by Spearman correlation analyses. Then, we calculated a linear step-wise regression model with the number of IRLs as the dependent variable and hemolytic parameters as independent variables adjusted for sex, age, DMT and disease duration. The same model was used with MRI parameters (total brain volume, total GM and cortical volume, WM volume, total lesion volume) as dependent variables and hemolytic parameters as independent variables adjusted for sex, age, and disease duration. To test the level of agreement of hemolysis parameters analyzed at different time points, the latter were compared as median values using Friedman's related-samples two-way analysis of variance by ranks with a clinical (relapse)- and radiological (Gd-enhancement)-based subanalysis. Intra-individual variance of hemolysis parameters was calculated using Bland-Altman method.

A value of *p* < 0.05 was considered statistically significant. All multiple analyses were corrected using Bonferroni method.

## Results

Seventy-five pwMS (53.3% female, 76.0% relapsing-remitting MS) were included with a mean age of 37.0 (SD 9.0) years and a median disease duration of 6 years (IQR 2–12), a median EDSS of 2.0 (IQR 1–3.5) and a median MSSS of 3.05 (IQR 0.99–5.85). Detailed demographics and characteristics are given in Table 1. IRL patients showed significantly higher scores in EDSS (*p* = 0.017) and MSSS (*p* = 0.036) compared to non-IRLs. Patients with IRLs were more commonly prescribed H-DMT (27; 57.4%) compared to patients without IRLs (11; 39.3%) (*p* = 0.024). Thirty-one (41.3%) pwMS experienced a relapse during the observation period (median time to MRI 7 weeks [IQR 4–19]), with 29 (38.7%) pwMS experiencing a severe relapse. No other relevant differences in demographics and clinical characteristics between non-IRL and IRL were found. In the control group, 43 patients (51.2% female) with a mean age of 38.3 years (SD 9.8) were included.

**Table 1:** Characteristics of pwMS.

	**pwMS (*n* = 75)**	**Non-IRL patients (*n* = 28)**	**IRL patients (*n* = 47)**	***p*-value**
**Demographic and clinical data**				
Female^a^	40 (53.3)	18 (64.3)	22 (46.8)	0.142
Age (years)^b^	37.0 (9.0)	35.5 (8.3)	38.0 (9.4)	0.255
Disease duration (years)^c^	6 (2–12)	4 (2.3–8.8)	8 (2–13)	0.180
Clinical activity^a^	31 (41.3%)	10 (35.7%)	21 (44.7%)	0.446
Severe relapse	29 (38.7%)	10 (35.7%)	19 (40.4%)	
EDSS^c^	2.0 (1–3.5)	1.3 (0–2.9)	2.3 (1.1–4)	0.017
MSSS^c^	3.05 (0.99–5.85)	2.39 (0.53–3.23)	3.91 (1.72–5.87)	0.036
RRMS^a^	57 (76.0)	24 (85.7)	33 (70.2)	0.128
**DMT** ^**a**^				
No DMT	11 (14.7)	2 (7.1)	9 (19.1)	0.024
M-DMT	26 (34.7)	15 (53.6)	11 (23.4)	
IFN	3 (4.0)	3 (10.7)	0 (0.0)	
Glatiramer acetate	7 (9.3)	4 (14.3)	3 (6.4)	
Dimethyl fumarate	14 (18.7)	8 (28.6)	6 (12.8)	
Teriflunomide	2 (2.7)	0 (0.0)	2 (4.3)	
H-DMT	38 (50.7)	11 (39.3)	27 (57.4)	
Fingolimod	13 (17.3)	2 (7.1)	11 (23.4)	
Natalizumab	4 (5.3)	2 (7.1)	2 (4.3)	
Alemtuzumab	6 (8.0)	2 (7.1)	4 (8.5)	
Rituximab	12 (16.0)	5 (17.9)	7 (14.9)	
Cladribine	3 (4.0)	0 (0.0)	3 (6.4)	
**MRI data**				
No. of IRLs^c^	1 (0–4)	NA	3 (1–9)	NA
No. of total lesions^c^	22 (11–45)	16 (8–49.8)	24 (11–45)	0.266
No. of IRLs/No. of total lesions^c^	0.07 (0.00–0.18)	NA	0.15 (0.08–0.32)	NA
Gd-enhancement^a^,^†^	15 (20.0)	3 (10.7)	12 (25.5)	0.101
Absolute brain volume (ml)^c^	1,090.9 (1,000.2–1,183.9)	1,096.3 (1,015.9–1,211.9)	1,077.4 (994.9–1,179.6)	0.385
Absolute GM cortical volume (ml)^c^	517.3 (473.0–560.6)	521.3 (473.0–567.9)	514.7 (471.3–560.6)	0.776
GM total volume (ml)^c^	667.7 (603.6–709.9)	671.5 (604.9–723.1)	666.5 (603.6–709.9)	0.648
Absolute WM volume (ml)^c^	422.3 (384.3–479.5)	427.5 (397.3–461.7)	417.6 (373.2–489.3)	0.373
Total lesion volume (ml)^c^	1.1 (0.4–3.6) [0.1–50.1]^d^	0.8 (0.2–3.6) [0.1–38.1]^d^	1.2 (0.5–3.8) [0.1–50.1]^d^	0.612
Absolute ventricular CSF volume (ml)^c^	346.5 (303.4–383.6)	344.3 (303.9–377.4)	348.65 (299.3–387.3)	0.798

*CSF, cerebrospinal fluid; DMT, disease-modifying treatment; EDSS, Expanded Disability Status Scale; MSSS, Multiple Sclerosis Severity Scale; GM, gray matter; IRL, iron rim lesion; NA, not applicable; pwMS, patients with multiple sclerosis; RRMS, relapsing-remitting multiple sclerosis; WM, white matter.*

a *8Number and percentage.*

b *Mean and standard deviation.*

c *Median and interquartile range.*

d *Range.*

† *In two pwMS, no contrast was administered*.

### Number of IRLs

**Figure 1:**
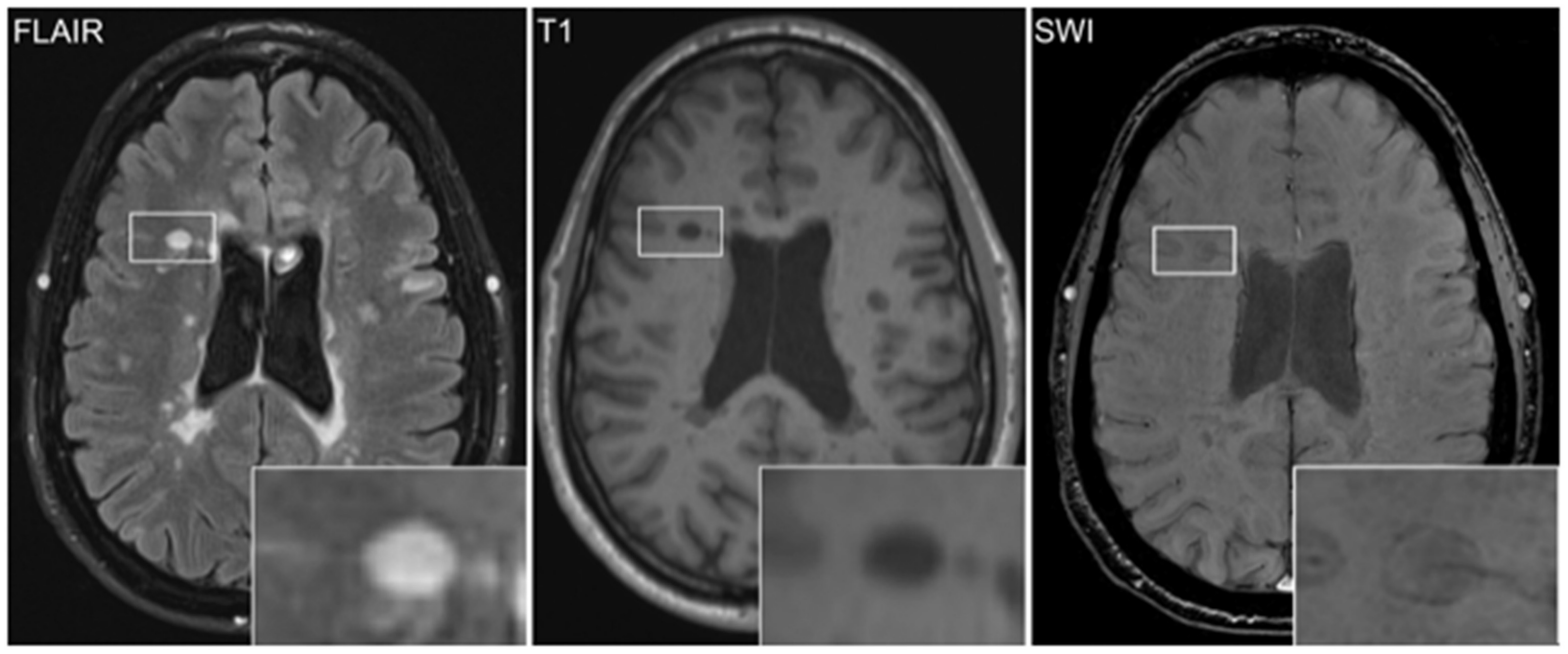
Iron rim lesions (IRLs) can be visualized by MRI *via* susceptibility-weighted imaging (SWI) as a hypointense rim of iron-laden microglia and macrophages surrounding the FLAIR-hyperintense lesion. Increased T1 hypointensity of the IRL indicates severe tissue destruction.

Among all patients, 281 IRLs were identified (Figure 1). Median number of IRLs in pwMS was 1 (IQR 0–4), 28 (37.3%) patients had no IRLs. Among those, 10 patients (nine females, mean age 36.9 years [SD 8.9], median disease duration of 3.5 years [IQR 1.0–8.3]) experienced a severe relapse. Two of those 10 patients showed Gd-enhancing lesions in the observed MRI. Only one patient had a SWI-sequence in the follow-up MRI. This patient did not show IRL formation within 1.5 years. Therefore, no predictors for conversion to IRLs could be identified. Number of IRLs correlated significantly with disease duration (*r*_s_ = 0.239, *p* = 0.039), EDSS (*r*_s_ = 0.387, *p* < 0.001) and MSSS (*r*_s_ = 0.289, *p* = 0.014) but not with patients' age (*r*_s_ = 0.151; *p* = 0.194).

### Hemolysis Parameters

**Figure 2:**
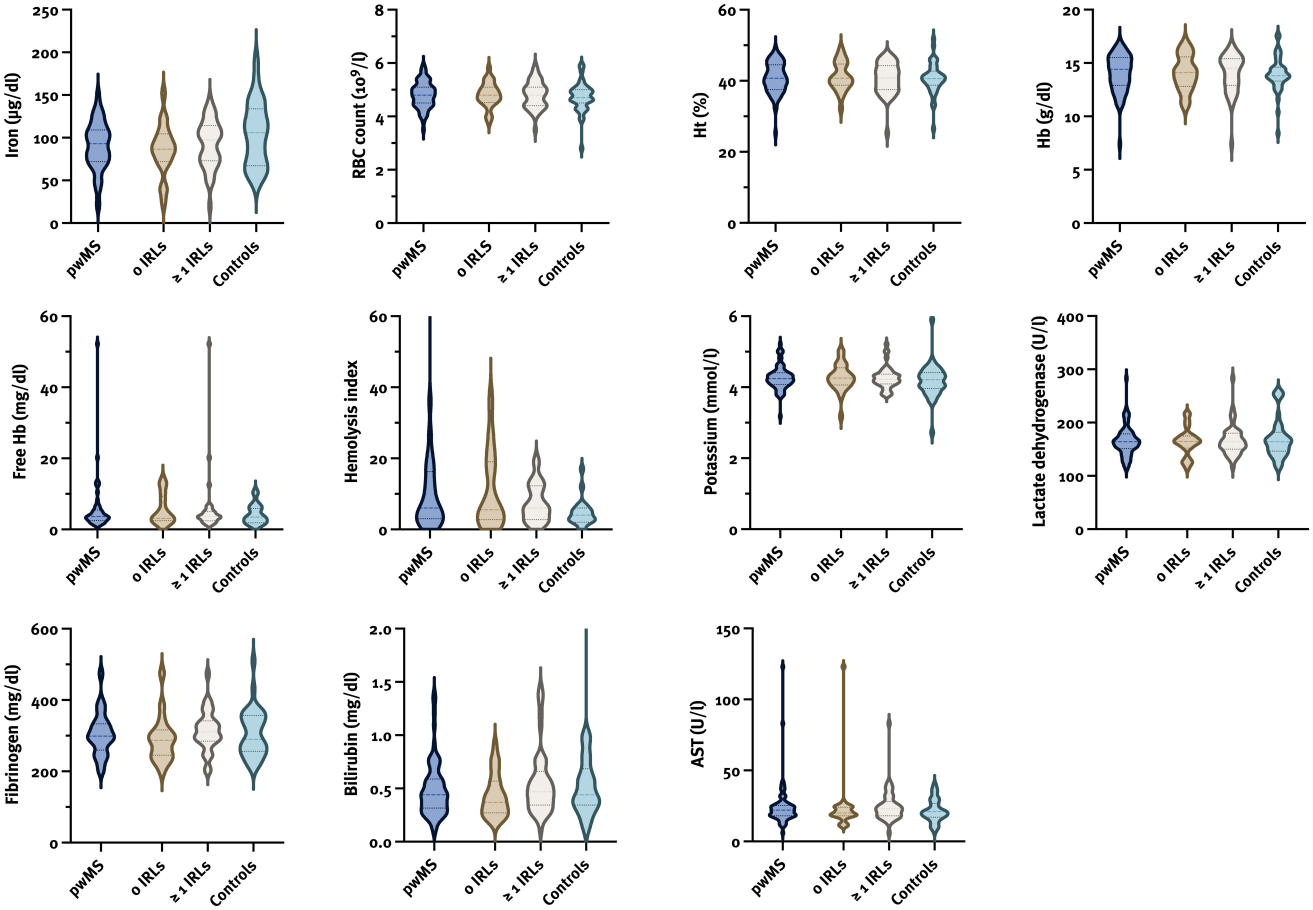
Violin plots for hemolysis parameters in pwMS according to the presence of IRLs and controls. AST, aspartate transaminase; Hb, hemoglobin; Ht, hematocrit; IRL, iron rim lesion; pwMS, patients with multiple sclerosis; RBC, red blood cell.

We analyzed the median values of hemolysis parameters in pwMS according to the presence of IRLs (no IRLs vs. ≥1 IRLs) (Figure 2; Supplementary Table 1) with gender (male with/without IRLs vs. female with/without IRLs) and disease course (relapsing vs. progressive MS) -related subanalysis, and compared them to those of controls; however, no differences were found. Besides, no correlation between the number of IRLs and hemolysis parameters was seen (Supplementary Table 1).

Furthermore,no significant correlation between hemolysis and clinical parameters was found. However, absolute brain volume was associated with fibrinogen (*β*= −0.34; 95% CI −1.32, −0.145; *p* = 0.016), and absolute cortical and total GM volumes were associated with Hb (*β* = 0.34; 95% CI 3.39, 24.68; *p* = 0.011 and *β* = 0.33; 95% CI 3.29, 28.95; *p* = 0.015; respectively).

**Figure 3:**
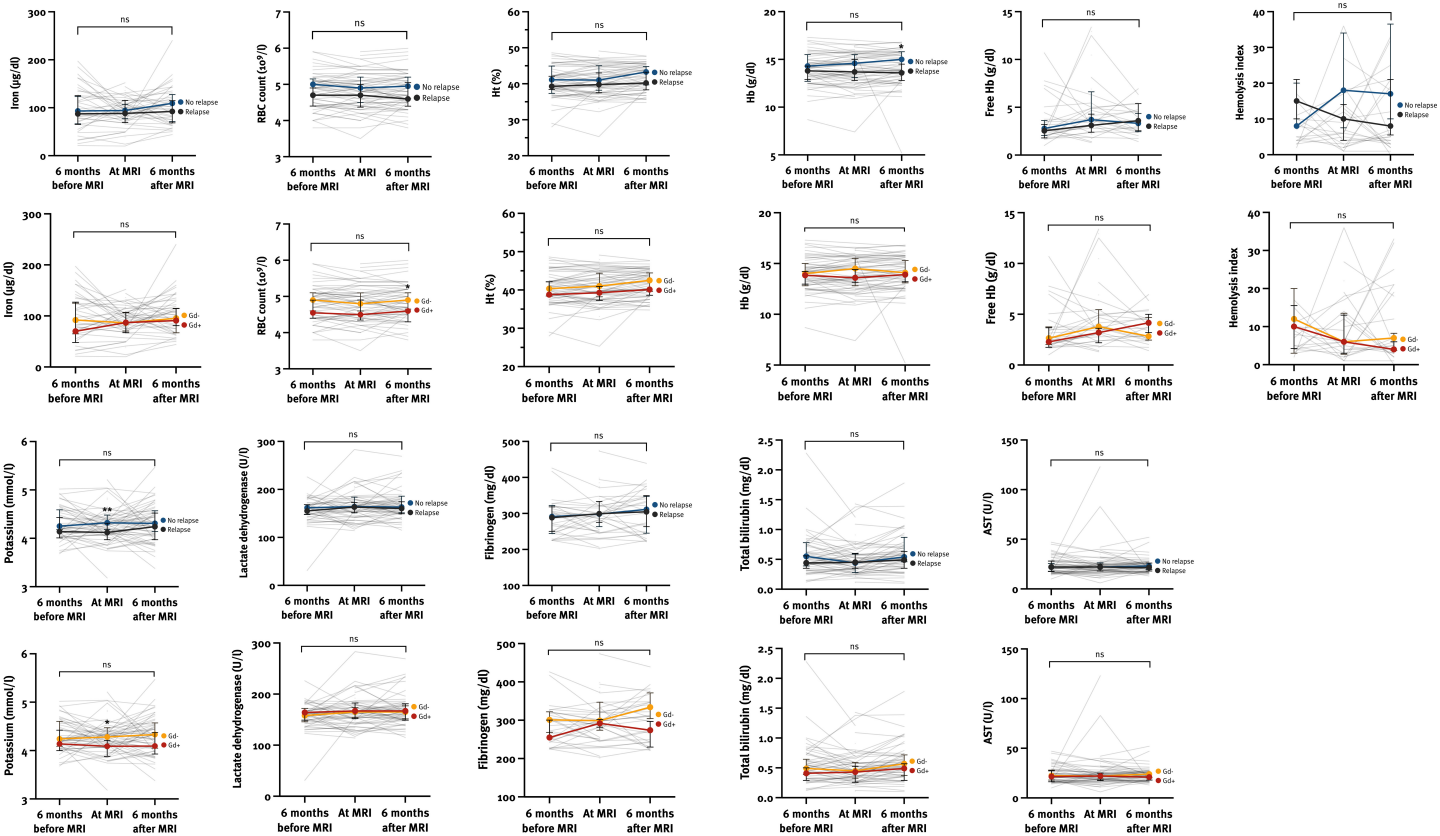
The variability of hemolysis parameters at different time points according to the clinical and radiological activity within the observation period. AST, aspartate transaminase; Gd, gadolinium-enhancing lesion; Ht, hematocrit; Hb, hemoglobin; ns, not significant; RBC, red blood cells. *p < 0.05, **p < 0.01 calculated by Mann-Whitney *U*-test.

We also analyzed the variability of hemolysis parameters in the period ±6 months from MRI on both a population and individual level (Supplementary Table 2; Figure 3). As 29/31 (93.5%) of pwMS experienced a severe relapse, no subanalysis based on the relapse severity was performed. The hemolysis parameters remained stable during the observation period regardless of clinical and radiological activity. However, pwMS with a relapse had a lower median Hb 6 months after MRI (13.6 [IQR 12.7–14.6] vs. 14.9 [IQR 13.7–15.8], *p* = 0.012) and a lower median potassium level at MRI (4.12 [IQR 3.97–4.33] vs. 4.35 [IQR 4.18–4.47], *p* = 0.006), and pwMS with at least one Gd-enhancing lesion had a lower median RBC level 6 months after MRI (4.6 [IQR 4.3–4.9] vs. 4.9 [IQR 4.6–5.2], *p* = 0.030) and a lower median potassium level at MRI (4.09 [IQR 3.87–4.27] vs. 4.28 [IQR 4.13–4.42], *p* = 0.029).

## Discussion

IRLs are currently being evaluated as imaging biomarkers of chronic activity and their potential future role in therapy monitoring, particularly in progressive MS. Since pwMS with IRLs compared to those without IRLs have a more severe disease course and transit earlier to a progressive stage, it is clinically relevant to find out associated, and perhaps even facilitating, factors for the presence of IRLs. Erythrocyte instability and low grade hemolysis seem to lead to increased free Hb in patients with systemic inflammation and immune activation (32), which has been also associated with the rate of brain atrophy in pwMS (35). Free Hb might enter the brain during active relapses or in the course of the low-grade increase of BBB permeability in progressive MS (43). After its degradation, free iron could contribute to neurodegeneration by amplifying oxidative injury and propagating proinflammatory activation of macrophages and microglia (8, 9). In our study we, thus, analyzed whether there is a direct association between hemolysis and the presence of IRLs in the MS brain.

Our study results confirm that a higher number of IRLs is associated with both longer disease duration and higher disability measured by EDSS, and this was independent of age. Higher EDSS in IRL patients was not associated with a significantly higher relapse activity, at least within 6 months from MRI, compared to non-IRL patients. The distribution of DMT also reflected a more severe clinical course in IRL patients as 57.4% of patients with IRLs received H-DMT, whereas 60.7% of patients without IRLs received no DMT or M-DMT. DMT was not changed within the time period of ±6 months from MRI. In addition, there was a trend for lower brain volume and higher lesion volumes in IRL patients compared with non-IRL patients consistent with recent literature (13, 15, 16)These data further support the view that iron rim lesions are markers for the progression of brain damage, but not for disease activity. Furthermore, IRLs could also serve as a marker for an earlier decision for H-DMT.

Apart from that, our retrospective cross-sectional study of 75 pwMS is the first to assess whether patients with IRLs have elevated levels of peripheral hemolysis parameters compared to patients without IRLs. Tested blood parameters for hemolysis were not significantly related in our cohort to the presence of IRLs nor with disease course or clinical (relapse) and MRI activity (Gd-enhancement). Despite the absence of a relation between hemolysis parameters and iron rims, MRI parameters (brain volume, GM volume) were associated with fibrinogen and Hb, being in line with a recently published study confirming an association between free Hb and brain atrophy in secondary progressive MS (35). However, several confounding factors may explain this possible association, including an older age of patients with progressive MS, thus being characterized by other comorbidities (e.g., diabetes, atherosclerosis, etc.). It is currently not known whether iron enters the macrophages predominantly *via* erythrocytes or free Hb and/or other forms of iron. Since the results show that hemolysis and IRLs are not significantly associated, the accumulation of iron-containing macrophages forming the iron rim around slowly expanding lesions cannot be explained by a continuous leakage of erythrocytes, Hb or iron through a weakly impaired BBB in the chronic phase of the disease. The fact that macrophages in the iron rim are not progressively loaded with iron but slowly and gradually loose signal intensity fits with the known long-term stability of iron rims observed by MRI (4, 44).

Our observation that there was no significant association between hemolysis and clinical (relapse) and MRI activity (Gd-enhancement) should be taken with caution, as in our study the median time between relapse and MRI was 7 weeks. This may have underestimated the number of patients with Gd^+^ lesions, as an open BBB is expected only for 4–6 weeks after relapse onset. However, a possible association between hemolysis parameters and disease activity should be analyzed in a young patient cohort with higher activity, a short disease course and a relapse-related MRI. Since it can be assumed that iron accumulation in the brain depends on the extent of the BBB opening, Gd-enhancing active lesions in patients with early MS might show a significant association with peripheral hemolysis in contrast to pwMS with long disease duration with a low burning chronic inflammation behind an only weakly impaired BBB.

The binding and transport system of iron itself in the CNS is complex and plays a crucial role in iron accumulation in the brain. Thus, it was recently indicated that iron accumulation in the CNS is simply the end stage of many different processes, reflected by altered expressions of different molecules involved in iron influx, efflux and storage, as well as iron sensors (28). Furthermore, not only the failure of iron transport but also inadequate antioxidant defense mechanisms of oligodendroglia and neurons compared with astrocytes influence the extent of iron-induced tissue damage (45–47). In addition, phagocytic cell instability due to long-term iron storage may further lead to cell dystrophy and death, resulting iron release and the propagation of oxidative damage. All of these points underscore the importance of investigating dysfunctional mechanisms of iron transport in a disease like MS. This knowledge may reveal new therapeutic targets to stop the vicious cycle of iron-induced CNS tissue damage. A first hint in this direction may be the observation of decreasing iron content of rim lesions per year in patients treated with dimethyl fumarate compared to patients treated with glatiramer acetate (48). In addition, dimethyl fumarate but not glatiramer acetate reduced inflammatory activity and associated iron levels in human microglia (49). Yet, further studies are necessary to evaluate therapeutic effects on IRLs and the long-term consequences for brain tissue.

The strengths of our study are the detailed characterization of the study cohort provided by the high-quality data from the Vienna MS database and the high-quality standard of MRI scans. The number of IRLs was counted manually by two experienced raters, providing low level of data variability. However, there are some limitations to this study. First, the retrospective and cross-sectional design as well as the relatively small sample size carry inherent potential of bias. Secondly, we did not have a quantitative threshold for partial IRL selection. However, since the inter-rater agreement was 98.7%, we can assume that partial IRLs were reliably detected. Further, ferritin, transferrin and total iron binding capacity, important biomarkers of iron status, could not be analyzed as these parameters were not performed within clinical routine. However, a recently published study found no correlation between different activation stages of MS with ferritin, transferrin, transferrin receptor and soluble transferrin receptor, as well as hepcidin, an important regulator of systemic iron homeostasis (25). Besides, the blood samples were drawn in the median time of 2 weeks before and after the MRI, which might present a less precise picture of the actual state in blood parameters during MRI acquisition. Nevertheless, iron rims are a stable feature with only slow changes over time. Moreover, we also analyzed the variability of hemolysis parameters in the 6-month period before and after MRI, which remained stable at both population and individual levels regardless of disease activity. It should also be noted that not only iron accumulation, but also myelin loss and perilesional white matter are reported to play a role in MR frequency or QSM image contrast (44). Since these considerations are particularly important for the quantitative interpretation of MR frequency or QSM data, and in our study the presence of iron rims was only assessed qualitatively (present or not), we do not see this as an interference with our results.

In conclusion, we did not find a significant association between peripheral hemolysis and IRL presence, which predominantly occur as a subtype of chronic active lesions in the progressive phase of MS behind an already almost completely closed BBB. However, hemolysis is confirmed to play a role in relation to the brain volume, which is the predominant feature in progressive MS. Further studies are needed to clarify the role of hemolysis in young and early diagnosed active pwMS with Gd-enhancing lesions indicating a wide-open BBB.

## Data Availability Statement

The raw data supporting the conclusions of this article will be made available by the authors, without undue reservation.

## Ethics Statement

The studies involving human participants were reviewed and approved by the Ethics Committee of the Medical University of Vienna (EC 1599/2021). Written informed consent for participation was not required for this study in accordance with the national legislation and the institutional requirements.

## Author Contributions

NK: acquisition of data, data management, statistical analysis and interpretation of data, and drafting of manuscript. GB, GK, TZ, BK, TB, FL, PR, HL, and SH: acquisition of data, interpretation of data, and critical revision of manuscript for intellectual content. AD-B: study concept and design, acquisition of data, interpretation of data, study supervision, and critical revision of manuscript for intellectual content. All authors contributed to the article and approved the submitted version.

## Conflict of Interest

NK has participated in meetings sponsored by, received speaker honoraria or travel funding from Celgene/BMS, Merck, Novartis, Roche and Sanofi-Genzyme, and held a grant for a Multiple Sclerosis Clinical Training Fellowship Programme from ECTRIMS. GB has participated in meetings sponsored by, received speaker honoraria or travel funding from Biogen, Celgene/BMS, Lilly, Merck, Novartis, Roche, Sanofi-Genzyme and Teva, and received honoraria for consulting Biogen, Celgene/BMS, Novartis, Roche, Sanofi-Genzyme and Teva. He has received financial support in the past 12 months by unrestricted research grants Celgene/BMS, Novartis. GK has participated in meetings sponsored by, received speaker honoraria or travel funding from Biogen, and received honoraria for consulting from Biogen. TZ has participated in meetings sponsored by or received travel funding from Biogen, Merck, Novartis, Roche, Sanofi-Genzyme and Teva. BK has participated in meetings sponsored by, received speaker honoraria or travel funding from Biogen, Celgene/BMS, Janssen, Merck, Novartis, Roche, Sanofi Genzyme and Teva, and received honoraria for consulting Biogen, Celgene/BMS, Merck, Novartis, Roche, Sanofi-Genzyme and Teva. TB has participated in meetings sponsored by and received honoraria lectures, advisory boards, consultations from pharmaceutical companies marketing treatments for MS: Allergan, Allmiral, Bayer, Biogen, Bionorica, Celgene/BMS, GSK, Janssen-Cilag, MedDay, Merck, Novartis, Octapharma, Roche, Sandoz, Sanofi-Genzyme, Teva. His institution has received financial support in the past 12 months by unrestricted research grants Biogen, Bayer, Celgene/BMS, Merck, Novartis, Sanofi Aventis, Teva and for participation in clinical trials in multiple sclerosis sponsored by Alexion, Bayer, Biogen, Merck, Novartis, Octapharma, Roche, Sanofi-Genzyme, Teva. FL has participated in meetings sponsored by and received honoraria lectures, advisory boards, consultations from pharmaceutical companies marketing treatments for MS: Actelion, Allmiral, Bayer, Biogen, Celgene, MedDay, Merck, Novartis, Octapharma, Roche, Sanofi-Genzyme, Teva-Ratiopharm. PR has received honoraria for consultancy/speaking from AbbVie, Allmiral, Alexion Astra Zeneca, Biogen, Merck, Novartis, Roche, Sandoz, Sanofi Genzyme, has received research grants from Amicus, Biogen, Merck, Roche. HL has received honoraria for lectures from Novartis, Biogen, ROCHE, Merck and Sanofi Aventis. SH has participated in meetings sponsored by or received speaker honoraria or travel funding from Biogen and Sanofi-Aventis. AD-B position as junior group leader for Translational Morphology in Neuroscience was supported by a research grant from Biogen. She has participated in meetings sponsored by, received speaker honoraria or travel funding from Biogen, Celgene BMS, Merck, Novartis, Roche and Sanofi; and has received an unrestricted grant from Merck GmbH, an affiliate of Merck KGaA.

## Publisher's Note

All claims expressed in this article are solely those of the authors and do not necessarily represent those of their affiliated organizations, or those of the publisher, the editors and the reviewers. Any product that may be evaluated in this article, or claim that may be made by its manufacturer, is not guaranteed or endorsed by the publisher.
