# Supplementary material for: Peripheral Hemolysis in Relation to Iron Rim Presence and Brain Volume in Multiple Sclerosis
Source: Front Neurol. 2022 Jun 29;13:928582. doi: 10.3389/fneur.2022.928582 (PMC9295598; doi:10.3389/fneur.2022.928582)
Supplement: Supplementary file 1 [file Data_Sheet_1.pdf]

Supplementary Table 1. Hemolysis parameters in pwMS according to the presence of IRLs and controls.

**Hemolysis parameter**[a]	**pwMS (n=75)**	**Non-IRL patients (n=28)**	**IRL patients (n=47)**	***p*-value**[b]	**Controls**	**p-value**[c]	**Spearman correlation coefficient (r_s)**	***p*-value**[d]
RBC (10^9/l)	4.8 (4.5–5.1)	4.8 (4.5–5.1)	4.8 (4.4–5.1)	0.930	4.7 (4.5–5.0)	0.768	–0.015	0.895
Reticulocytes	48.6 (41.0–59.5)	†	51.0 (39.0–65.1)	NA	†	NA	0.696	0.125
Ht (%)	40.7 (37.5–44.5)	40.6 (38.4–44.7)	40.8 (37.5–44.3)	0.861	40.6 (38.8–42.7)	0.991	–0.051	0.665
Hb (g/dl)	14.4 (12.9–15.5)	14.1 (12.8–15.6)	14.5 (12.9–15.4)	0.900	13.8 (13.3–14.6)	0.843	–0.011	0.922
Free Hb (mg/dl)	3.64 (2.51–5.46)	3.14 (1.48–9.30)	3.65 (2.42–5.01)	0.969	3.49 (1.90–5.90)	0.905	0.083	0.594
Hemolysis index	6 (3–16.3)	5.5 (2.8–19)	6 (2.8–13.3)	0.839	4 (2–6)	0.146	0.052	0.793
Potassium (mmol/l)	4.24 (4.08–4.41)	4.26 (4.06–4.55)	4.22 (4.09–3.37)	0.665	4.21 (3.96–4.41)	0.413	–0.056	0.643
Total bilirubin (mg/dl)	0.44 (0.32–0.59)	0.37 (0.26–0.56)	0.46 (0.35–0.61)	0.052	0.44 (0.35–0.69)	0.154	0.191	0.103
Lactate dehydrogenase (U/l)	164 (151–178.8)	164.5 (152–174.8)	164 (149.5–180)	1.000	164 (146.5–181.8)	0.992	–0.017	0.890
Fibrinogen (mg/dl)	398.5 (259.8–333)	287 (245.3–316.3)	310.5 (284.5–341.8)	0.084	289.5 (256.0–356.8)	0.266	0.214	0.144
AST (U/l)	22 (18–25.3)	20 (18–24)	23 (18–28.8)	0.099	21 (17–26.8)	0.141	0.068	0.561
Iron (µg/dl)	93 (72–109)	86.5 (72–104.5)	97.5 (73–114.3)	0.434	106 (67.3–133.8)	0.273	0.154	0.232

AST: aspartate transaminase, Hb: hemoglobin, Ht: hematocrit, IRL: iron rim lesion, NA: not applicable, pwMS: patients with multiple sclerosis, RBC: red blood cells

[a]Median and interquartile range, [b]p-value for comparison of non-IRL and IRL patients calculated by Mann-Whitney U-test, [c]p-value for comparison of non-IRL, IRL patients and controls calculated by Kruskal-Wallis test, [d]p-value for Spearman correlation analyses comparison of parameters in pwMS

[†]Sample size too small to calculate distributions

Supplementary Table 2. The level of agreement of hemolysis parameters in pwMS according to the clinical and radiological activity within the observation period using Friedman's related-samples two-way analysis of variance by ranks.

**Hemolysis parameter[a]**	**6 months before MRI**	**At MRI**	**6 months after MRI**	**6 months before MRI**	**At MRI**	**6 months after MRI**	***p*-value**
	**Relapse (n=31; 10 non-IRL, 21 IRL)**			**No relapse (n=44; 18 non-IRL, 26 IRL)**			
RBC (10^9/l)	4.7 (4.4–5.0)	4.7 (4.3–5.0)	4.6 (4.3–5.1)	5.0 (4.4–5.2)	4.9 (4.5–5.3)	5.0 (4.6–5.2)	ns
Reticulocytes	†	†	†	†	51 (39–65.1)	†	NA
Hematocrit (%)	39.3 (38.4–42.1)	39.8 (37.5–43.2)	40.2 (38.3–43.6)	41.1 (37.3–45.7)	41.0 (38.2–45.3)	43.3 (39.7–45.1)	ns
Hemoglobin (g/dl)	13.8 (12.9–14.3)	13.7 (12.6–15.1)	13.6 (12.7–14.6)	14.3 (12.7–15.8)	14.6 (13.1–15.7)	15.0 (13.6–15.8)	ns
Free hemoglobin (mg/dl)	2.54 (1.71–4.37)	3.11 (2.35–4.55)	3.59 (2.43–5.43)	2.78 (2.04–4.27)	3.70 (3.07–6.76)	3.32 (2.43–4.48)	ns
Hemolysis index	7.5 (5–13.5)	5 (1.5–10)	4 (2.3–17.5)	4 (3.3–10)	9 (3–17)	8.5 (4.5–20.5)	ns
Potassium (mmol/l)	4.14 (4.01–4.46)	4.12 (3.97–4.33)	4.24 (3.94–4.52)	4.25 (4.18–4.59)	4.32 (4.18–4.47)	4.31 (4.09–4.56)	ns
Total bilirubin (mg/dl)	0.44 (.34–0.57)	0.45 (0.34–0.59)	0.49 (0.32–0.66)	0.55 (0.37–0.78)	0.44 (0.28–0.61)	0.54 (0.46–0.93)	ns
Lactate dehydrogenase (U/l)	155 (145–170)	163 (149.8–172.8)	160 (148.5–175)	161.5 (147.3–166.8)	164 (151–185)	163 (151–185.5)	ns
Fibrinogen (mg/dl)	288 (248–320.8)	299 (274.5–336.5)	304.5 (241–366.5)	291 (243.5–322.8)	298 (251–333)	311 (244–347)	ns
AST (U/l)	22 (17–28)	22 (18–25)	21.5 (17.8–26.3)	22 (17.8–27.3)	22 (18–26)	23 (19.5–25.3)	ns
Iron (µg/dl)	87 (57.5–133.5)	88 (67.5–118)	92 (65–125)	93 (65–134)	94 (74.5–107.8)	109 (71–130)	ns
	**Gd-enhancement n=15; 3 non-IRL, 12 IRL)**			**No Gd-enhancement (n=58; 25 non-IRL, 33 IRL)[‡]**			

RBC (10^9/l)	4.6 (4.4–4.9)	4.5 (4.3–4.9)	4.6 (4.3–4.9)	4.9 (4.5–5.1)	4.9 (4.5–5.2)	4.9 (4.6–5.2)	ns
Reticulocytes	†	†	†	†	51 (45.2–65.1)	†	NA
Hematocrit (%)	38.8 (38.3–42.3)	39.3 (37.3–41.2)	40.1 (37.7–40.8)	40.9 (37.6–44.1)	41.2 (37.6–44.9)	42.8 (38.7–44.8)	ns
Hemoglobin (g/dl)	13.9 (12.9–14.3)	13.6 (13.0–14.4)	13.9 (12.9–14.2)	14.1 (12.8–15.3)	14.5 (12.9–15.7)	14.5 (12.9–15.7)	ns
Free hemoglobin (mg/dl)	2.28 (1.25–5.87)	3.20 (1.90–3.97)	4.16 (2.01–5.90)	2.63 (1.98–3.93)	3.78 (2.69–3.67)	3.19 (2.43–4.55)	ns
Hemolysis index	10 (2.8–16.5)	6 (2–19)	4 (3–7)	6 (4–9.8)	6 (3–13.5)	6 (3–21)	ns
Potassium (mmol/l)	4.14 (3.89–4.45)	4.09 (3.87–4.27)	4.10 (3.87–4.45)	4.23 (4.10–4.59)	4.28 (4.13–4.42)	4.32 (4.07–4.58)	ns
Total bilirubin (mg/dl)	0.41 (0.28–0.61)	0.43 (0.25–0.57)	0.49 (0.29–0.59)	0.54 (0.38–0.74)	0.45 (0.33–0.62)	0.57 (0.37–0.77)	ns
Lactate dehydrogenase (U/l)	164 (139.5–176)	167 (149–183)	167 (145–181.5)	160.5 (147.3–167)	164.5 (151–177.3)	161 (149.5–178)	ns
Fibrinogen (mg/dl)	255 (247–305)	292 (264–312)	274 (227.5–304)	301 (244–322)	299 (253–340)	317.5 (247.8–353)	ns
AST (U/l)	21.5 (15.8–29.8)	22 (17–26)	21 (18–25)	22 (18–28)	21.5 (18–25)	23 (18–26)	ns
Iron (µg/dl)	70 (40–162)	87 (61.3–108.3)	91 (76.5–125.8)	93 (65–134)	90 (72–107.5)	103 (66.5–130)	ns

AST: aspartate transaminase, Gd: gadolinium, IRL: iron rim lesion, NA: not applicable, ns: not significant, RBC: red blood cells

[a]Median and interquartile range

†Sample size too small to calculate distributions, ‡In two pwMS, no contrast was administered
